# Comment on “Intravitreal Ampicillin Sodium for Antibiotic-Resistant Endophthalmitis: *Streptococcus uberis* First Human Intraocular Infection Report”

**DOI:** 10.1155/2014/395480

**Published:** 2014-07-22

**Authors:** Luigi Toma, Enea Gino Di Domenico, Grazia Prignano, Fabrizio Ensoli

**Affiliations:** ^1^Department of Infectious Disease, San Gallicano Dermatology Institute, 00144 Rome, Italy; ^2^Istituto Pasteur-Fondazione Cenci Bolognetti, Department of Biology and Biotechnology Charles Darwin, Sapienza University, 00185 Rome, Italy; ^3^Department of Clinical Pathology and Microbiology, San Gallicano Dermatology Institute, 00144 Rome, Italy

In the paper by Velez-Montoya et al. [1], the authors reported the first description of a case of intraocular infection in humans caused by an antibiotic-resistant strain of* Streptococcus uberis*.

We would like to point out that the absence of any description of the method used for bacteria identification in this paper raises some concerns related to the possibility of a misidentification of this bacterium as a pathogen affecting the human eye.

*S. uberis* is an environmental pathogen responsible for a high proportion of cases of clinical and subclinical mastitis in ruminant and nonruminant species [2]. The nutritional flexibility associated with an assortment of metabolic options allows* S. uberis* to occupy a discrete ecological niche [3]. Some studies have hypothesized that the flexibility of this bacterium under various environments and conditions might possibly favour infection also in humans [4, 5]. However, the evidence and putative role of* S. uberis* as a human pathogen are very limited and the methods used for the identification are frequently questionable [6].

In fact, phenotypic bacterial identification by commonly used systems such as Vitek, Facklam scheme, and similar conventional methods has been generally employed. However, in most cases of supposed human infections by* S. uberis* these techniques showed a low level of accuracy [6, 7]. Facklam described a case of human infection where all the isolates, previously classified as* S. uberis,* have been subsequently identified as* Globicatella sanguinis* [7] and a consistent body of evidence supports the notion that one of the most recurrent mistakes in the identification of gram-positive cocci, using phenotypic bacterial identification methods, is represented by the lack of distinction between* S. uberis* and* Enterococcus* spp. [8, 9].

A conventional scheme for the identification of* S. uberis* strains isolated from bovine milk samples and based on 11 biochemical tests also showed 6% frequency of misidentifications between* S. uberis* and* Enterococcus faecalis* [10]. On the other hand, infections caused by* E. faecalis* are largely described in the literature [11–17].* E. faecalis* is known to represent a virulent pathogen frequently associated with endophthalmitis with very poor clinical prognosis [14, 18]. Endophthalmitis caused by* E. faecalis* has been described in a diabetic patient after biliary surgery [19], while other reports described ocular infections after cataract extractions [20–22]. Recently Bains et al. and Tang et al. also reported the emergence of endophthalmitis caused by* E. faecium* vancomycin-resistant strains [23, 24]. Indeed, the intraocular infections caused by* E. faecium* previously described in the literature are not in contrast with the image reported in Figure 1(b) of the paper by Velez-Montoya et al. [1].

In conclusion the phenotypic bacterial identification systems have been repeatedly found to fail the classification of* E. faecalis* on behalf of* S. uberis*. Thus, in our opinion the absence of any detailed description of the technique used for the bacterium identification in the paper by Velez-Montoya et al. [1] raises some concern since the method of identification may affect the validity and reliability of the diagnosis.

Therefore we consider some information from the authors necessary regarding the description of the methods used for the identification, particularly considering that this might represent the first case of human intraocular infection caused by* S. uberis* and also in consideration that the pathogenic potential of this bacterium in humans is still under debate.
